# Profiles of transcriptome and metabolic pathways after hypobaric hypoxia exposure

**DOI:** 10.1186/s12953-022-00198-y

**Published:** 2022-09-24

**Authors:** Jin Xu, Wen-jie Chen, Zhan Wang, Ming-yuan Xin, Shen-han Gao, Wen-jing Liu, Kai-kun Wang, Jing-wei Ma, Xin-zong Yan, Yan-ming Ren

**Affiliations:** 1 Qinghai University, Xining, 810001 China; 2 Qinghai University Clinical Medicine Class 6, Grade 20, Xining, 810001 China; 3 Department of Biomedical Engineering, School of Medicine, Tsinghua University, Beijing, 100084 China; 4 Qinghai University Clinical Medicine Class 1, Grade 19, Xining, 810001 China; 5 Qinghai University, Plateau Biology Jing Ying Class, 19 Grade, Xining, 810001 China; 6 Qinghai University the Graduate Student of Foundation Medical of 2020, Xining, 810001 China; 7 Qinghai University Clinical Medicine Class 3, Grade 20, Xining, 810001 China; 8 Qinghai University Clinical Medicine Class 1, Grade 19, Xining, 810001 China; 9 Qinghai University the Graduate Student of Foundation Medical of 2021, Xining, 810001 China; 10 Qinghai University, Xining, 810001 China

**Keywords:** Lipid metabolism, Hypobaric hypoxia, Microarray, PPARA, ANGPTL4

## Abstract

**Background:**

Hypoxia is a risk factor for non-alcoholic fatty liver diseases, leading to permanent imbalance of liver lipid homeostasis and steatohepatitis. However, a detailed understanding of the metabolic genes and pathways involved remains elusive.

**Methods:**

In vivo experiments were designed to analyze body weight and lipid metabolism changes of rats under hypoxia. After this, we combined microarray analysis and gene overexpression experiments to validate the core mechanisms involved in the response to hypoxia.

**Results:**

The hypobaric hypoxia treated rats exhibited significantly increased serum triglycerides (TG) (*p* < 0.05), despite no significant changes in serum alanine aminotransferase (ALT) and blood glucose (BG) were observed. In addition, serum high-density lipoprotein cholesterol (HDL-C) greatly increased after 3 days and then returned to normal level at 30 days. Interestingly, serum low-density lipoprotein cholesterol (LDL-C) showed an opposite pattern. Transcriptome analysis, qRT-PCR, ICC revealed that the genes PPARA, ANGPTL4, CPT-I, ACC and LPL play a crucial role in response to hypobaric hypoxia. IPA pathway analysis further confirmed that PPARA-mediated regulation of ANGPTL4 participated in TG clearance and lipoprotein metabolism. Finally, the PPARA-ANGPTL4 pathway was validated in rats and HL 7702 cells treated with Fenofibrate, a PPARA specific agonist.

**Conclusions:**

Our study showed this pathway plays an important role on lipid metabolism caused by hypobaric hypoxia and the potential target genes associated with oxygen-dependent lipid homeostasis in the liver.

**Supplementary Information:**

The online version contains supplementary material available at 10.1186/s12953-022-00198-y.

## Background

Oxygen is crucial to the metabolic process. A state of hypoxia, characterized by a lack of adequate levels of oxygen, activates a variety of complex pathways at both the cellular and organ level to reinstate oxygen homoeostasis. The liver is essential to metabolism and extremely sensitive to low-oxygen conditions [1]. Lipid accumulation in the blood vessels and other liver tissues leads to an insufficient blood supply to the hepatocytes and causes lipid peroxidation, setting up a hypoxic microenvironment. Prolonged exposure to hypoxia can promote uncontrolled lipid accumulation, and lipid metabolism has been closely associated to various diseases with growing importance. Recent studies demonstrated high-altitude-associated hypobaric hypoxia plays an important role in the regulation of lipid metabolism [2]. In particular, HDL-C, LDL-C, and triglycerides are key participants in lipid metabolism and strongly associated with cardiovascular risks [3, 4]. Preliminary studies showed increasing HDL-Cand reducing LDL-C as altitude increases. In accordance, Mohanna et al. [5] studied high-altitude populations and demonstrated their HDL-C and triglyceride levels were elevated, while their total cholesterol remained normal and their LDL-C levels were low. By profiling data in a young group of newcomers being chronically exposed for 8 months to 3550 m, Siques et al. [6] found that total cholesterol levels did not change but observed a mild decrease of LDL-C and a rise in triglycerides. Praveen Vats et al. [7] reported HDL-C levels increased significantly on high-altitude exposure in two different ethnic groups. However, recent studies showed a different picture. Siques et al. [8] found total cholesterol, LDL-C, VLDL-C and triglycerides greatly increased in rats after 15 days of exposure, while HDL-C levels decreased. These contradictory results suggest an urgent need to further study the relationship between lipid metabolism and hypobaric hypoxia.

It is well known that the liver is a central organ that metabolizes glycogen and lipids and supplies energy-producing substrates to the peripheral tissues in order to maintain their function under different conditions. Interestingly, studies in the last decade have demonstrated that non-alcoholic fatty liver disease (NAFLD) is also closely associated with hypobaric hypoxia, which exerts its function by regulating lipid metabolism [9–13]. As to the underlying molecular mechanisms, recent reports suggest the pathological significance of hypoxia inducible factors (HIFs), master regulators of the hypoxic response, modulate their target genes to regulate cell energy metabolism [10, 12, 14]. The majority of target genes are key metabolic enzymes closely associated with lipid metabolism, including GLUT-1, LDHA, DEC1, DEC2 and ANGPTL3 [14]. On the other hand, some studies shed light on the NAFLD mechanism, which is mediated by PETN and the NF-*k*B pathway. PTEN and NF*-k*B promote hepatocyte fat accumulation by regulating IRS-PI3K-PDK1-Akt pathway under hypoxia [9, 13]. Specifically, PTEN dephosphorylates the second messengers generated by the activation of PI3K (phosphoinositide 3-kinase), thereby down-regulating or terminating insulin signaling downstream PI3K. However, the precise molecular mechanism linking NAFLD and hypobaric hypoxia remains uncertain.

Variation in lipid metabolism caused by hypobaric hypoxia involves the activation of a number of key enzymes and metabolic pathways. Peroxisome proliferator-activated receptor alpha (PPARA) is a nuclear receptor protein and a central regulatory hub impacting lipid metabolism in the liver. A genome-wide scan in Tibetans revealed PPARA is significantly associated with the expression of several genes encoding proteins that control fatty acid metabolism. Further studies demonstrated that PPARA increased plasma levels of non-esterified fatty acids, suggesting a possible role in the regulation of lipid metabolism due to high-altitude hypobaric hypoxia. Interestingly, angiopoietin-like proteins (ANGPTLs) are one of most of important regulators of lipid metabolism and were identified as highly sensitive PPAR target genes. Notably, ANGPTL4 can be cleaved into N- and C-terminal fragments that have different functions. Specifically, nANGPTL4 is known to function as a lipid metabolism regulator by inhibiting lipoprotein lipase (LPL) activity and stimulating lipolysis of the white adipose tissue (WAT), which results in increased levels of plasma triglycerides (TG), LPL, HL-dependent hepatic cholesterol uptake and fatty acids. These studies demonstrated ANGPTL4 is the main participant in lipid metabolism caused by high-altitude hypobaric hypoxia. However, the underlying molecular mechanisms and specific role of the PPARA-ANGPTL4 pathway regulating lipid metabolism remain elusive.

To shed further light on this problem, we combined animal experiments with microarray techniques to identify changes in lipid metabolism under hypobaric hypoxia. Data analysis identified the function of PPARA, ANGPTL4 and other novel genes regulating lipid metabolism and downstream metabolic pathways. Here, we show that hypobaric hypoxia induced changes in various lipid metabolism components. The levels of PPARA-regulated expression of ANGPTL4 play an important role mediating lipid metabolism. The enrichment different metabolic pathways at different stage in lipid metabolism demonstrates this process is more complex than previously thought.

## Materials and methods

### Cell culture

Human liver cells HL-7702 (ATCC) were cultured in DMEM/Ham’s F12 (v/v, 1:1) supplied with 10% FBS, 100 U/ml penicillin, and 100 μg/ml streptomycin at 37 °C in a humidified atmosphere of 95% air and 5% CO_2_. The hypobaric hypoxia cell model was performed by incubating the culture under 1% O_2_, 94% N_2_, and 5% CO_2_ conditions. The cells were sealed and placed at 37 °C for 24 h, and after this harvested in RAPA lysis buffer and TRIZOL for downstream analyses.

### Hypobaric hypoxia rats model

Sprague-Dawley male rats (purchased from Genechem Co. Ltd., Shanghai) were randomly allocated to 7 groups (10 animals per group) containing normal rats (L), rats exposed to chronic hypobaric hypoxia for 3, 15, and 30 days, and rats exposed to chronic hypobaric hypoxia and fenofibrate (PPAR agonist) for 3, 15 and 30 days. Rats in the chronic hypobaric hypoxia groups were exposed to a simulated altitude atmosphere of 5500 m (380 mmHg), which was implemented using a FLYDWC50-1C low pressure hypoxic experimental cabin (Guizhou Fenglei Air Ordnance LTD, Guizhou, China). During breeding and experimental procedures, animals in both groups were housed in the same density per cage at a controlled ambient temperature of 25 ± 2 °C and 50 ± 10% relative humidity under a 12 h light/12 h dark cycle. Rats were given standard rodent chow and water ad libitum. Following overnight fasting, the rats were sacrificed using a 10% chloral hydrate anesthetic (0.4 ml/100 g body weight, IP). The right robe of the liver was snap-frozen in liquid nitrogen and then stored at − 80 °C for further analyses. Control rats (L) were anesthetized and sacrificed on day 1 and processed in a similar fashion as described above. The research protocol was approved by the Institutional Animal Care and Use Committee at the Qinghai University School of Medicine (Xining, China). All animal experiments followed national or institutional guidelines for the care and use of animals.

### RNA preparation and quality control

Total RNA from HL-7702 cells was extracted using Trizol and purified using the RNeasy RNA extraction kit. Quality control of extracted RNA was subsequently examined by both Thermo Nanodrop 200 and Agilent 2100 bioanalyzer using the Agilent RNA 6000 Nano Kit. Quality RNA was subjected to microarray analysis until meeting the following criteria: 1.7 < A260 / A280 < 2.2 by Thermo Nanodrop 200 and RIN > =7 and 28S/18S > 0.7 by Agilent 2100 bioanalyzer.

### Microarray processing and data analysis

A total of 6 GeneChip microarrays (Affymetrix 901,838) were hybridized with 3 pairs of samples to determine the gene expression profiles of control and treatment (chronic hypobaric hypoxia for 24 hours) HL-7702 cell samples according to the manufacturer’s instructions. Briefly, qualified total RNA was firstly subject to poly(A) tailing (37 C for 15 min) and biotin ligation (25 C for 30 min) using the FlashTag Biotin HSR labeling Kit before its hybridization with the microarray gene chips (48 C, 60 rpm, 16–18 h) in the GeneChip Hybridization Oven 645. After hybridization, the Genechip Hybridization Wash and Stain Kit was used to wash and stain the array chips on a GeneChip Fluidics Station 450. Finally, microarrays were scanned using the GeneChip scanner 3000 and data and cell files obtained from GCOS 1.1. The expression raw data was imported to R (www.r-project.org) and analyzed in the Bioconductor *affy* package (www.bioconductor.org). Logarithmic (base 2) intensity measures were obtained by RMA. Intensity was converted to nonlogarithmic values and rescaled by adjusting the mean intensity on each array to 400.

The gene expression profile was preprocessed using the *Limma* package in Bioconductor and Affymetrix annotation files. The Background correction, quantile normalization and probe summarization of the microarray data were performed using the Robust Multi-array average algorithm to obtain the gene expression matrix.

### Identification of differential expressed genes (DEGs) and pathway analysis

The *Limma* package was used to normalize the microarray raw data. By executing the LIMMA package, the differentially expressed genes (DEGs) were filtered, and the functional enrichments were consequently performed [15]. The genes with |log2FC| > =2 and *P* < 0.05 were considered as significantly different between the control and treatment groups.

The list of significant genes identified using the Affymetrix probe set IDs, fold changes and *p*-values were uploaded into the Ingenuity Pathway Analysis (IPA) tool (www.ingenuity.com). Each clone identifier was mapped to its corresponding gene object in the Ingenuity Pathway Knowledge Base (IPKB). These focus genes were then used for constructing biological networks, using the “IPA” core analysis function. To start building the networks, the application queries the IPKB for interactions between focus genes and all other gene objects stored in the knowledge base. Every resulting gene interaction has supporting literature findings available online. IPA then computes a score for each network according to the fit of the user’s set of significant genes. The score is derived from a *p*-value and indicates the likelihood of the focus genes that are present in a given network being found together as a result of random chance. A score of 2 indicates that there is a 1-in-100 chance that the focus genes randomly appear together in a network. Therefore, scores of 2 or higher have a probability of at least 99% of not being generated by chance.

### Plasma and hepatic lipid profiles

Plasma lipids were measured on Days 0, 15 and 30. The plasma triglycerides (TG), total cholesterol (TC), Low-density lipoprotein (LDL), High-density lipoprotein (HDL), and Very-low density lipoprotein (VLDL) were measured using the Vitros DT60 II Chemistry System (Johnson & Johnson, Minnesota, MN). The liver samples were homogenized using a Stir-Pak®, (Barrington, Il). T-Chol and TG were extracted in a chloroform-methanol mixture (2:1) and measured with the same Vitros DT60 II Chemistry System.

### Data analysis and statistics

The results were entered into a database and analyzed using SPSS 22.0 (SPSS, Inc., Chicago, Ill, U.S.A.). The mean, standard deviation, standard error, and confidence intervals were calculated for each parameter. The normality was established using the Kolmogorov–Smirnov test, and the distributions of all parameters were found to be normal. Statistical analysis of the differences between the two conditions was performed using a *t*-test of independent variables, and analysis of variable measurements across time was performed using a *t*-test of related variables. Pearson’s correlation coefficients were also estimated. The results were considered significant when the *p*-value was lower than 0.05.

## Results

### Biochemical parameters and lipid profiles

**Fig. 1:**
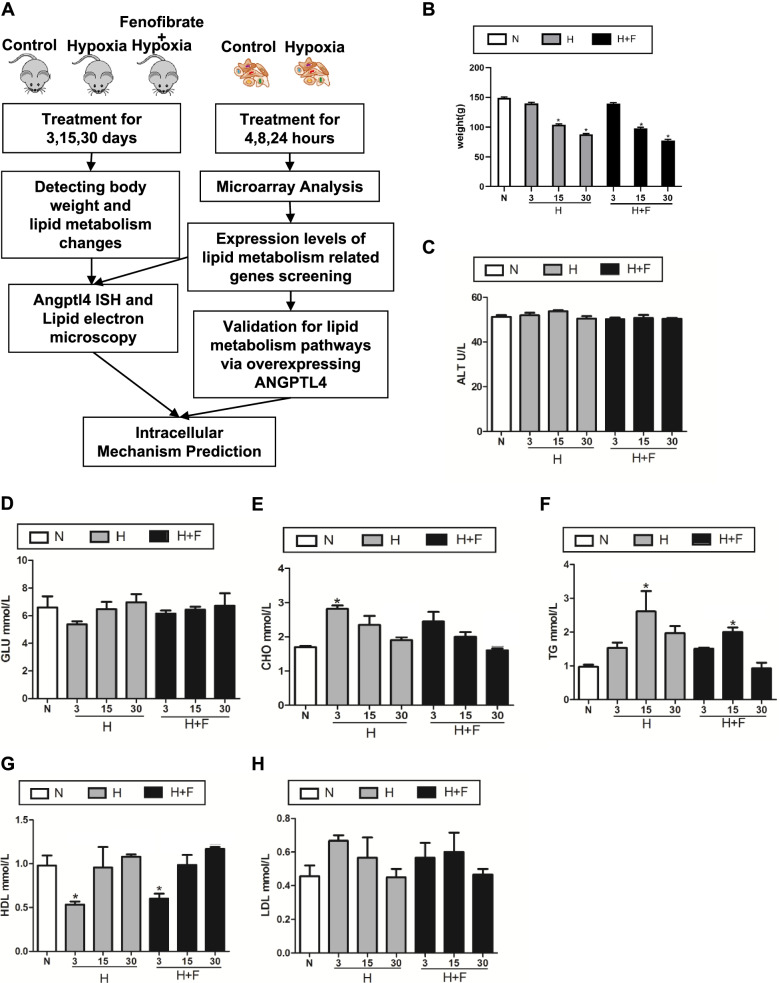
Effects of the Hypobaric Hypoxia treatment on rat biochemical parameters. **A** Schematic diagram of the hypobaric hypoxia rat and cell model. **B-H**, different biochemical parameters after hypobaric hypoxia and Fenofibrate treatment. There is an observable increase in CHO, TG and LDL after hypobaric hypoxia treatment. At the same time, the parameters weight, GLU and HDL significantly decrease. ALT: Serum alanine aminotransferase, GLU: blood glucose, TG: serum triglycerides, CHO: total cholesterol, LDL: low-density lipoprotein, HDL: high-density lipoprotein. H: Hypobaric hypoxia treatment, H + F: Hypobaric hypoxia and Fenofibrate treatment. Student’s *t*-test, paired tail, * < 0.05

To explore the hypobaric hypoxia influence on lipid metabolism, we first built a hypobaric hypoxia animal model using Sprague-Dawley male rats, following previous studies. Serum alanine aminotransferase (ALT) and blood glucose (BG) were not significantly changed after exposure to hypobaric hypoxia (Fig. 1C and D). However, serum triglycerides (TG), total cholesterol (TC), low-density lipoprotein (LDL), and high-density lipoprotein (HDL) were significantly affected by hypobaric hypoxia treatment. Interestingly, cholesterol and TG had the highest levels (*p* < 0.05) after exposure for 3 days, and were restored to normal levels on day 30 (Fig. 1E and F). At the same time, HDL showed an opposite trend and decreased on day 3, after which it increased gradually to normal levels on day 30 (Fig. 1G). The LDL levels also increased, with the largest effects being evident on day 3 (*p* < 0.05) (Fig. 1H), which is consistent with a previous study [16].

It has been well established that PPARA and ANGPTL4 are important regulators of lipid metabolism [17, 18]. To assess whether the PPARA-ANGPTL4 pathway also regulates lipid metabolism caused by hypobaric hypoxia, we applied Fenofibrate, a PPARA specific agonist, to evaluate changes in biochemical parameters and lipid profiles (Fig. 1A). Our results showed that the PPARA agonist relieved the effects caused by hypobaric hypoxia on lipid metabolism (Fig. 1 B-H). Importantly, lipid profile results demonstrated that hypobaric hypoxia plays an important role in lipid metabolism and that different lipid components change dynamically at different stages of hypobaric hypoxia. The PPARA-ANGPTL4 pathway may be central regulator of lipid metabolism.

### Overview of microarray data

**Fig. 2:**
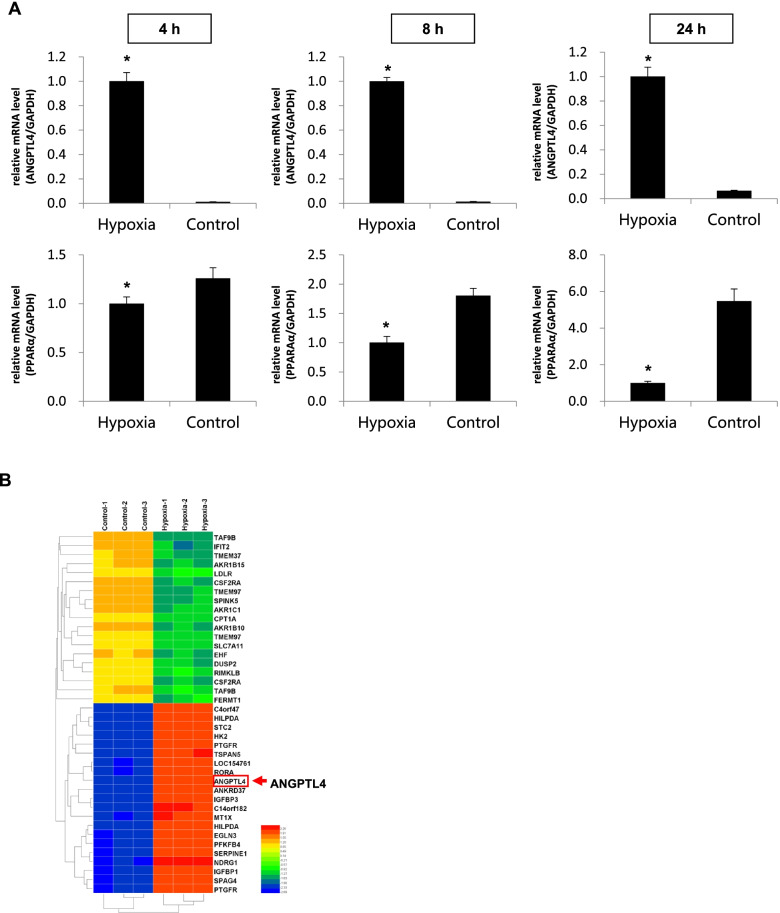
Differentially expressed genes after Hypobaric Hypoxia treatment in HL-7702. **A** and** B** Relative mRNA and protein expression levels of PPARA and ANGPTL4 after hypobaric hypoxia treatment. PPARA was down-regulated after hypobaric hypoxia treatment and inhibited mostly at 24 h. ANGPTL was up-regulated after hypobaric hypoxia treatment and expressed at the highest level at 8 h. **C** Heatmap of differentially expressed genes after hypobaric hypoxia treatment. We obtained 528 and 765 significantly down- and up-regulated genes, respectively, in the hypobaric hypoxia treated samples. The heatmap results demonstrated our data has high reproducibility. The red frame represents the genes up-regulated by ANGPTL4

To test whether PPARA and ANGTL4 are involved in regulating lipid metabolism during hypobaric hypoxia, we built a hypobaric hypoxia model using the liver cell line HL-7702. We evaluated the expression levels of both PPARA and ANGTL4 at 4 h, 8 h, and 24 h. The qRT-PCR results demonstrated ANGPTL4 and PPARA are significantly up- and down-regulated after hypobaric hypoxia treatment, respectively (Fig. 2A and B). These observations are consistent with previous studies [18, 19] and support the idea that the PPARA-ANGPTL4 pathway plays a role on lipid metabolism. We further investigated the detailed mechanism behind lipid metabolism associated with hypobaric hypoxia by scanning differential metabolic genes and pathways in across the entire genome. Considering the importance of the liver in lipid metabolism, including its association with lipid biosynthesis, lipoprotein secretion, and cholesterol transport, we exposed a normal human liver cell line (HL-7702) to hypobaric hypoxia for 24 hours. This allowed us to identify the key genes regulating lipid metabolism using microarrays (Fig. 1A). We detected a total of 49, 395 transcripts in our microarray data and an almost perfect correlation between the different replicates (Fig. 2A). Microarray analysis identified differentially expressed genes with log_2_FC in expression levels (*p* < 0.05). We combined the replicated data and identified 528 and 765 genes with significantly down-regulation and up-regulation, respectively, in the hypobaric hypoxia treated samples (Fig. 2B and Table S1). Furthermore, we obtained a larger number of genes related to lipid metabolism, including *CPT1*, *ANGPTL4*, and *LPL*. Notably, we found PPARA expression levels are significantly decreased after hypobaric hypoxia treatment (*p* < 0.01), while ANGPTL4 shows a different trend (Fig. 2C). These results indicate PPARA and ANGPTL4 may be key regulators of metabolism caused by hypobaric hypoxia.

### Pathways involved in lipid metabolism due to hypobaric hypoxia

**Fig. 3:**
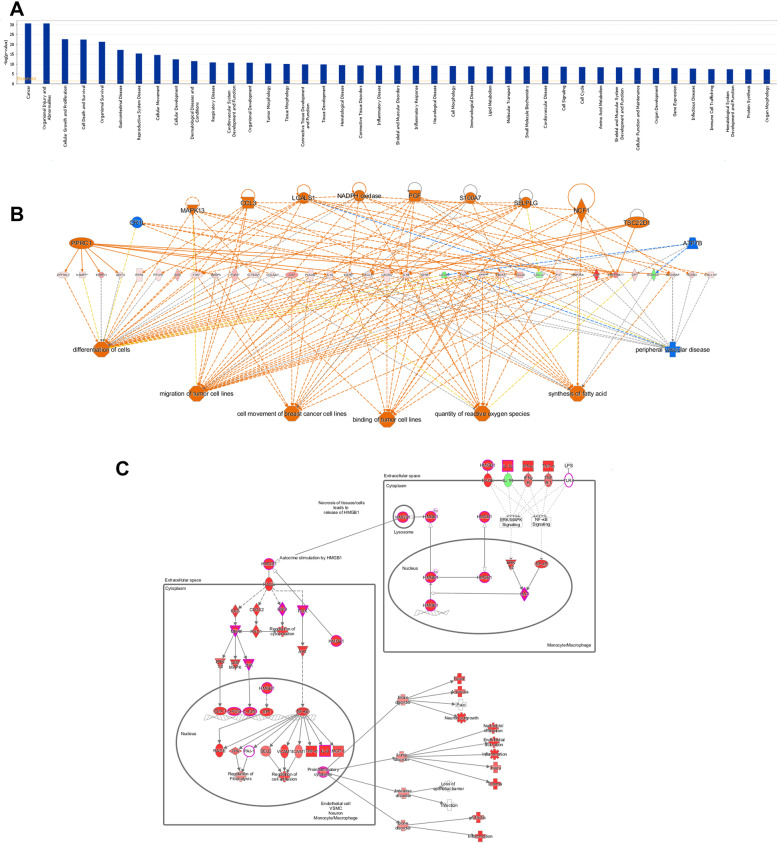
Ingenuity pathway analysis (IPA) and enriched pathways for differentially expressed genes after Hypobaric Hypoxia treatment. **A** Differentially, expressed genes due to hypobaric hypoxia. Enriched pathways from the IPA results include cancer, immunity and lipid metabolism. **B** Regulation network obtained from the IPA results. **C** Detail of HMGB1 related pathway in the IPA results. Several immune response-related genes, such IL-1R, MAPK and ERK, are involved in the regulation of hypobaric hypoxia

The Hypobaric hypoxia-induced decrease in PPARA expression levels attenuates the inhibition of ANGPTL4 [20]. Accordingly, we aimed to investigate which novel pathways and genes may be involved in the lipid metabolism mediated by ANGPTL4. Hence, we analyzed differentially expressed genes using an ingenuity pathway analysis (IPA) and obtained numerous enriched pathways (Fig. 3A and Table S2), including cancer, cellular growth and proliferation (Fig. 3A and B), PPAR Regulation of Inflammatory Signaling, LXR/RXR Activation and β-adrenergic signaling. These enriched pathways were further observed to show ANGPTL4 is involved in lipid metabolism and might be, as previous studies proposed, an important cancer regulator [21–23]. Notably, we also observed a high number of novel pathways are seemingly associated with lipid metabolism. For example, interferon signaling, ErbB2-ErbB3 signaling, PPARα/RXRα activation, fatty acid synthesis, and NF-κB activation are shown in Fig. 3B. Importantly, we also identified an immune response pathway that is mediated by HMGB1 and associated with inflammation, cell differentiation and tumor cell migration. We hypothesize that a crosstalk between the PPARA-ANGPTL4 pathway and cancer related pathways occurs during the regulation of lipid metabolism. The enriched pathways obtained from the differentially expressed genes provide detailed information on the genes likely participating in lipid metabolism and advance our understanding of this process as caused by hypobaric hypoxia.

### PPARA-ANGPTL4 is key responder to hypobaric hypoxia stimulation

**Fig. 4:**
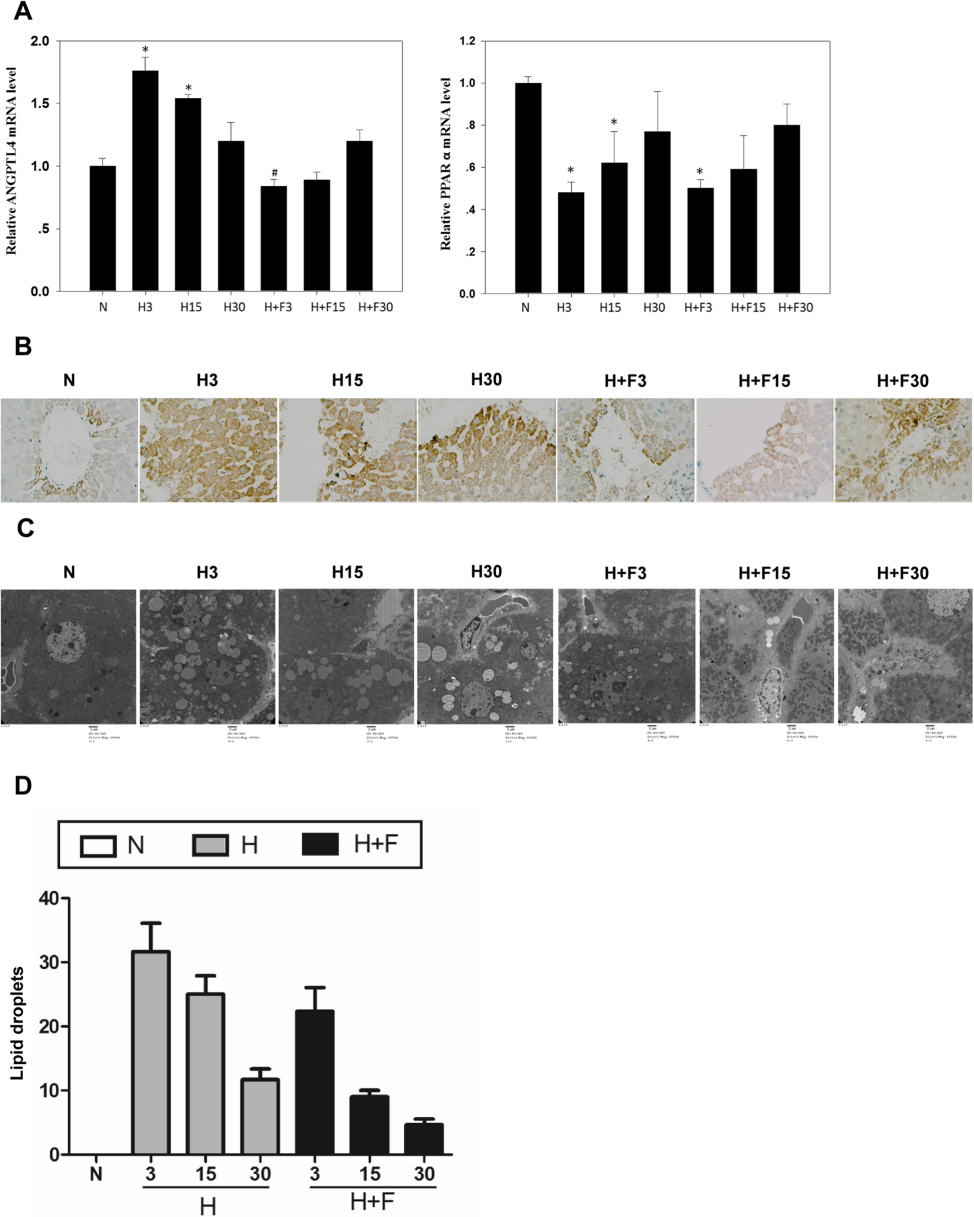
ANGPTL4 and PPARA are key regulators of lipid metabolism following Hypobaric Hypoxia. **A** The ANGPTL4 and PPARA relative mRNA levels in hypobaric hypoxia rats. ANGPTL4 was up-regulated to the highest level at day 3, while PPARA was down-regulated to the lowest level at day 3. This trend was abated by Fenofibrate, which is consistent with results obtained in HL-7702 cells. Student’s *t*-test, paired tail, * < 0.05. **B** Immunohistochemistry of ANGPTL4 in mouse liver tissues. ANGPTL4 was up-regulated due to hypobaric hypoxia treatment and inhibited by Fenofibrate. **C** Electron microscopic biopsy of rat livers in different groups. Hypobaric hypoxia treatment increases the amount of lipid droplets, while fenofibrate treatment significantly attenuates these changes due to the inhibition of ANGPTL4. **D** Statistical results for the lipid droplets. Hypobaric hypoxia treated rat livers contain a higher number of lipid droplets. This number decreases following fenofibrate treatment

To evaluate the role of PPARA-ANGPTL4 in the regulation of lipid metabolism, we first validated the expression levels of hypobaric hypoxia liver tissues by qRT-PCR. Our results are consistent with those observed in HL-7702 cell line sequencing data (Fig. 2A), with significant changes occurring on days 3 and 15 after hypobaric hypoxia treatment (Fig. 4A). We then tested the effects of PPARA and ANGPTL4 on key lipid components using fenofibrate, which is a PPARA agonist that activates PPARA and inhibits ANGPTL4 expression [24, 25]. The qRT-PCR results demonstrated ANGPTL4 is mostly down-regulated on day 3 post-fenofibrate treatment, and restored to normal levels on day 15 (Fig. 4A). In addition, PPARA mRNA levels did not significantly changed compared to the hypobaric hypoxia group without fenofibrate treatment (Fig. 4A). Immunohistochemistry staining on rat liver tissues also confirmed hypobaric hypoxia treatment significantly increased the expression of ANGPTL4, and that subsequent treatment with fenofibrate inhibited ANGPTL4 by activating PPARA (Fig. 4B).

We further investigated the influence of ANGPTL4 inhibition on lipid metabolism. Rats treated with fenofibrate showed a reduced body weight, TG, TC, HDL and LDL compared with the hypobaric hypoxia treated group (Fig. 1). Electron microscopic biopsy on rat livers showed an increase in the amount of lipid droplets in the hypobaric hypoxia treated group. Treatment with fenofibrate significantly attenuated these changes due to the inhibition of ANGPTL4 (Fig. 4C and D). These results support the idea that PPARA-ANGPTL4 are differentially regulated by hypobaric hypoxia and mediate lipid metabolism.

**Fig. 5:**
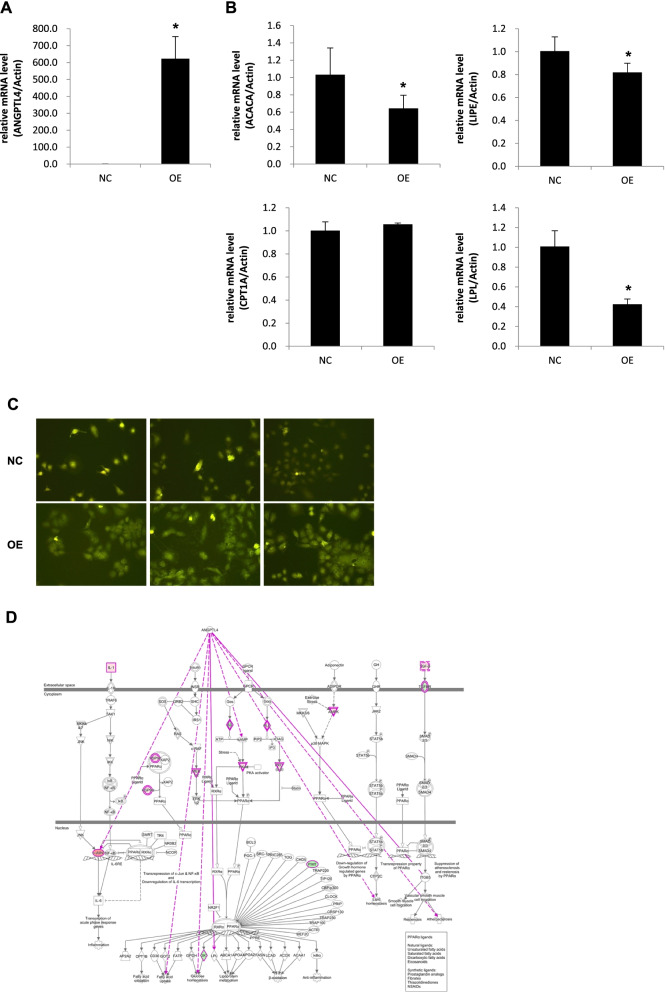
Overexpression of ANGPTL4 has an equal effect on the regulation of pathway genes and lipid metabolism caused by Hypobaric Hypoxia. **A** The overexpression of ANGPTL4 on mRNA levels. **B** Lipid metabolism-related genes relative mRNA levels after the expression of ANGPTL4, showing the up-regulation of ACACA, LIPE and LPL. Student’s *t*-test, paired tail, * < 0.05. **C** Oil red O staining assays validated the overexpression of ANGPTL4 regulates lipid metabolism by increasing the number of lipid droplets in treated cells compared to controls. **D** IPA predicted pathways based on qRT-PCR results. The predicted pathways are enriched in lipid metabolism, similar to the observations following hypobaric hypoxia treatment

We then tested whether PPARA-ANGPTL4 regulate downstream target genes mediating lipid metabolism by overexpressing ANGPTL4 in HL-7702 cells and measuring its influence on target genes. The qRT-PCR analysis revealed a 500-fold increase in mRNA levels compared with controls (Fig. 5A). ANGPTL4 target genes were significantly affected due to the expression of ANGPTL4 (Fig. 5B and S2). In particular, the expression of ACACA, LIPE and LPL, which are key regulators of lipid metabolism, was significantly downregulated after overexpressing ANGPTL4 (Fig. 5B). Furthermore, we also evaluated the influence of ANGPTL4 overexpression on lipid metabolism. The oil red O staining assays confirmed that ANGPTL4 overexpression regulates lipid metabolism by increasing the number of lipid droplets in treated cells compared to controls (Fig. 5C). We performed IPA based on qRT-PCR results after overexpressing ANGPTL4 and built a pathway revealing ANGPTL4 is central regulator of lipid metabolism (Fig. 5D). The overexpression of ANGPTL4 affected the key regulator of IL1 and the TGF-β pathways (Fig. 5D).

The above results demonstrate ANGPTL4 and PPARA respond to hypobaric hypoxia and play an important role in the regulation of lipid metabolism.

## Discussion

Dysregulation of lipid metabolism is increasingly recognized as a risk factor for many metabolic diseases, including nonalcoholic fatty liver and cardiovascular diseases. In addition, obstructive sleep apnea syndrome affects serum lipids [26–30] and previous studies have suggested hypoxia may influence lipid metabolism. Here, we showed that body weight decreases during hypobaric hypoxia treatment in rats, similar to previous studies in mice [31]. Moreover, we found that the levels of key serum components that are closely associated with lipid metabolism, such as TG, TC, LDL, and HDL, were also significantly affected. Interestingly, different lipid components exhibit dynamic and inconsistent changes during hypobaric hypoxia treatment. For example, TC and LDL significantly increased in the early stages of anoxic treatment (for 3 days), but the levels returned to normal at the end of treatment (for 30 days) (Fig. 1G and J). However, TG and HDL levels showed completely opposite trends (Fig. 1H and I). Previous reports on changes in HDL and LDL serum are inconsistent. Praveen Vats et al. [7] reported people undergoing longtime high-altitude hypoxia exhibited bodyweight loss and increased HDL levels. In contrast, Siques et al. [8] showed hypoxia induced VLDL, TG, and LDL serum reached maximum levels at 15 days while HDL levels were significantly downregulated. The latter results are consistent with our findings. The contradictory results of the above studies indicate the mechanisms behind lipid metabolic change after hypobaric hypoxia are complex. In fact, this process might include multiple metabolic pathways and potential mechanisms, which is of considerable significance to understand lipid metabolism and treat associated diseases.

Chronic intermittent hypoxia (CIH) induced lipid metabolism disorder has been reported in several studies, most of which mainly focused on the regulation of two important genes, PPARA and ANGPTL4 [16, 17, 19, 20, 31]. Under hypoxic conditions, the expression of PPARA was downregulated and led to a reduced inhibition of ANGPTL4. The upregulation of ANGPTL4 inhibits the activity of LPL, which in turn affects the synthesis and degradation of downstream metabolites, such as TG, HDL and LDL [16, 17, 19, 20, 32, 33]. However, studies on the molecular mechanism of hypobaric hypoxia-induced changes in lipid metabolism remain understudied, including the roles played by PPARA and ANGPTL4 in this process.

To further investigate the key signaling pathways and regulatory genes related to PPARA and ANGPTL4 in hypobaric hypoxia, we used qRT-PCR and microarrays to analyze changes in transcriptome expression before and after hypoxia treatment. We identified a large number of differentially expressed genes involved in lipid metabolism (Fig. 2D and Table S1). We note that the expression of PPARA significantly decreased after hypobaric hypoxia induction, while the expression of ANGPTL4 significantly increased (Figs. 2A, 4A and B). In subsequent experiments, we found that a PPARA agonist inhibited the ANGPTL4 signaling pathway and attenuated lipid metabolic changes. The overexpression of ANGPTL4 resulted in changes on multiple signaling pathways (Figs. 2D and 5B). The two experiments indicated that both PPARA and ANGPTL4 play important roles in hypobaric hypoxia-mediated lipid metabolism.

Microarray analysis identified a large number of differentially expressed genes associated with lipid metabolism, and the signal pathway enrichment analysis showed an enrichment in metabolic, immunity and cancer related pathways following hypobaric hypoxia. Interestingly, many cancer-related and immunity signaling pathways were also enriched, including cell growth and proliferation, cell death and survival (Fig. 3A), or the NF-*κ*B pathway (Fig. 3C). This is consistent with previous studies showing ANGPTL4 is involved in the regulation of cancer and immune related diseases [21, 23]. This also highlights the complexity of hypobaric hypoxia mediated regulation of ANGPTL4 on lipid metabolic diseases such as NAFLD, which is caused by an abnormal accumulation of fat in liver cells [9, 12, 14]. Previous studies focusing on key genes within the lipid metabolic pathway have provided few insights [10, 12]. Here, we identified a large number of target genes associated with PPARA-ANGPTL4. These genes may constitute novel targets for the treatment of hypobaric hypoxia related diseases.

In summary, this study unveiled the importance of PPARA and ANGPTL4 in hypobaric hypoxia-induced lipid metabolism, and identified several new regulatory signaling pathways that provide a new basis for future research and the treatment of related disease.

## Supplementary Information

**Additional file 1.**

**Additional file 2.**

## Data Availability

The datasets used and analyzed during the current study are available from the corresponding author on reasonable request.

## Abbreviations

ALT: Alanine aminotransferase
ANGPTLs: Angiopoietin-like proteins
BG: Blood glucose
CIH: Chronic intermittent hypoxia
DEGs: Differential expressed genes
HDL: High- density lipoprotein
HDL-C: High-density lipoprotein cholesterol
HIFs: Hypoxia inducible factors
IPA: Ingenuity Pathway Analysis
IPKB: Ingenuity Pathway Knowledge Base
LDL: Low-density lipoprotein
LDL-C: Low-density lipoprotein cholesterol
LPL: Lipoprotein lipase
NAFLD: Non-alcoholic fatty liver disease
PI3K: Phosphoinositide 3-kinase
PPARA: Peroxisome proliferator-activated receptor alpha
TC: Total cholesterol
TG: Triglycerides
VLDL: Very –low density lipoprotein
WAT: White adipose tissue
